# Research Hotspots and Trends in Ciliopathies: A Bibliometric and Visualization Analysis

**DOI:** 10.1155/bmri/9033339

**Published:** 2026-03-24

**Authors:** Qian Dong, Jinhao Zhu, Jiang Liu, Huan Xu

**Affiliations:** ^1^ Department of Nephrology, The Affiliated Lihuili Hospital of Ningbo University, Ningbo, China; ^2^ Department of Neurosurgery, The Shanghai Tenth People′s Hospital, School of Medicine, Tongji University, Shanghai, China, tongji.edu.cn

**Keywords:** ciliopathies, CiteSpace, primary ciliary dyskinesia, research trends, visualization analysis

## Abstract

**Purpose:**

This study conducted a comprehensive bibliometric analysis of research hotspots and development trends in ciliopathies to provide an overview of the global research landscape.

**Methods:**

The study retrieved relevant literature on ciliopathies from the Web of Science Core Collection up to July 27, 2024. Using the CiteSpace visualization software, co‐occurrence network analyses were performed on the authors, institutions, and keywords of the literature.

**Results:**

A total of 1821 papers were included, demonstrating a wave‐like increasing trend in annual publication volume. Among English‐language publications, Omran, Heymut had the highest number of articles. The cooperation network among the United States, the United Kingdom, and China was broader than that of other countries or regions. Omran, Heymut had the highest number of publications (64), placing him among the most prolific authors (defined as ≥ 60 publications); Knowles, Michael R. (58) and Leigh, Margaret W. (51) ranked second and third, respectively. Research hotspots mainly focused on functional mutations, congenital heart disease, cystic fibrosis, dyskinesia, primary ciliary dyskinesia, and genetics. Current ongoing hotspots include genetics, dynein, multiple morphological abnormalities, motile cilia, male infertility, variants, and sperm flagella.

**Conclusion:**

This study delineates the evolutionary trajectory of research themes, identifies established and emerging hotspots, and maps the global collaborative network. These findings provide a systematic, evidence‐based framework to guide future research directions, foster cross‐disciplinary collaboration, and optimize resource allocation in the ciliopathy field.

## 1. Introduction

Ciliopathies, as a type of genetic disease caused by structural and/or functional abnormalities of cilia, have emerged as one of the hot topics in the field of biomedical research in recent years [1, 2]. Cilia, as tiny and highly dynamic structures on the cell surface, not only participate in basic physiological processes, such as cell movement and sensing external signals, but also play a vital role in embryonic development, organ formation, and maintaining homeostasis of the body [3–5]. Therefore, abnormalities in ciliary function often lead to a variety of complex diseases, including kidney diseases, neurological disorders, skeletal abnormalities, and visual and auditory impairments, which seriously affect the quality of life of patients [6–9].

With the rapid development of high‐throughput sequencing technology and bioinformatics methods, the genetic basis of ciliopathies has been gradually revealed, leading to the discovery of numerous genes related to cilia formation, maintenance, and functional regulation. Studies have shown that disruption in cholesterol metabolism involving Dhcr7 and Insig1/2 can result in skeletal formation defects and changes in the homeostasis of primary cilia formation [10]. Other research has indicated that gap junction–mediated intercellular Ca^2+^ waves play an important role in the maintenance of motile cilia in the ependymal, suggesting that motile ciliopathies may be improved by enhancing functional gap junctions through pharmacological or genetic manipulation [11]. Additionally, it has been found that there is a close connection between the transcription factor SOX9 and cilium inhibition [12]. These findings not only deepen our understanding of the biological characteristics of cilia but also provide important foundations for the early diagnosis, genetic counseling, and precision treatment of ciliopathies [13]. However, the vast and rapidly expanding literature on ciliopathies presents a challenge for researchers to grasp the intellectual structure and evolutionary dynamics of the field. Traditional narrative reviews are often subjective and lack quantitative rigor. Therefore, there is an urgent need for a systematic, data‐driven analysis to objectively identify research frontiers, trace thematic shifts, and map the collaborative landscape. A review of existing literature reveals that while a limited number of bibliometric studies on ciliopathies exist, they are often constrained by factors such as a narrower timeframe, the use of a single analytical tool, or a lack of in‐depth quantification of collaborative networks. This study is aimed at addressing these gaps by providing an updated analysis extending to 2024, employing a multitool approach for cross‐validation and richer insights, and delivering a comprehensive quantitative assessment of global collaboration patterns and evolving research hotspots. This bibliometric review is aimed at filling this gap by providing a macroscopic overview that can inform strategic planning and pinpoint emerging niches for future investigation, thereby offering a specific advance by synthesizing a fragmented body of knowledge into a coherent roadmap.

Bibliometric and visualization analysis serve as a scientific research method that deeply explores and visualizes literature data through quantitative and qualitative means. It provides a clear reflection of the current research status, key issues, and future development trends in a particular field [14]. CiteSpace is a citation visualization analysis software used to measure and analyze scientific literature data. It can transform a large volume of literature into knowledge maps, allowing a more intuitive and visual understanding of a research area, which facilitates the rapid integration of key information and decision‐making [15]. In this study, CiteSpace was utilized to review and summarize the current status, hotspots, and emerging trends in ciliopathy research, providing a reference for subsequent research and application. Through a comprehensive analysis of English‐language literature on “ciliopathies” from the Web of Science (WOS) database, this study employed CiteSpace visualization software to analyze publication trends, author cooperation networks, institutional distribution, keyword co‐occurrence, clustering, timelines, and burst detection. These analyses comprehensively elucidate the research hotspots and development trends in the field of ciliopathies.

## 2. Materials and Methods

### 2.1. Data Sources and Search Strategy

The source of the literature for this study was the WOS database. The search strategy involved the use of both subject terms and free terms. A comprehensive search of the WOS Core Collection was conducted on July 27, 2024. The search strategy employed a topic search query designed to capture literature on ciliopathy, using a combination of key terms such as “kartagener syndrome,” “primary ciliary dyskinesia,” “ciliary motility disorder,” “immotile cilia syndrome,” and their variants. This search, which covered all years from the database inception to the search date, yielded an initial result of 3188 articles.

The literature screening was based on the following predefined criteria:

Inclusion criteria are as follows: (1) document type: research articles or reviews; (2) language: English; (3) content: focus on the etiology, diagnosis, treatment, or mechanism of ciliopathies; (4) publication year: 2000–2024.

Exclusion criteria are as follows: (1) nonresearch document types, such as conference abstracts, editorials, letters, and meeting proceedings; (2) preprints and retracted publications (retracted articles were identified and excluded based on the WOS “retracted” label, resulting in the exclusion of three records); (3) duplicate publications.

The construction of search terms for this study was based on the following principles: (1) core term coverage: the recognized umbrella term “ciliopathies” was employed; (2) inclusion of disease aliases and historical terminology: to comprehensively capture literature, key disease aliases and former designations such as “Kartagener syndrome,” “primary ciliary dyskinesia (PCD),” and “immotile cilia syndrome” were incorporated; (3) synonym expansion: based on the Medical Subject Headings (MeSH) and preliminary literature review, related expressions such as “ciliary motility disorder” and “ciliary dysfunction” were supplemented. This strategy is aimed at balancing retrieval sensitivity and specificity.

After applying these criteria and removing duplicates, 1821 papers were finally included in the study.

### 2.2. Data Processing

CiteSpace is a visualization software that operates in the Java environment. It was developed by Professor Chaomei Chen from Drexel University, United States, and it uses algorithms such as pathfinder networks to compute sample literature in a specific field. CiteSpace explores the potential value of the transformation of a discipline and the cutting‐edge trend of its future development by illustrating these through visualized maps. In this study, CiteSpace was employed to conduct a visualization analysis of literature in the field of ciliopathy research from the inception of the database to 2024, providing a reference for promoting related research in this domain. The literature from WOS was exported in plain text format, uniformly named as “download‐xx,” and then imported into CiteSpace 6.2.R4 for format conversion. Prior to analysis, a rigorous data cleaning process was implemented to ensure data quality. First, duplicates were removed using a two‐step process: initially via the WOS built‐in “remove duplicates” function (which matches records based on title, author, source, and publication year), followed by a second check using EndNote software to capture any remaining duplicates. A total of 1367 duplicate records were identified and removed. Second, the document types were strictly filtered to include only “article” and “review,” thereby excluding other types of publications such as conference abstracts, editorials, letters, and meeting proceedings. A manual verification of a random 10% sample of the excluded records was conducted to confirm the accuracy of this filtration. Finally, author and institution names were standardized to minimize ambiguity; author names were cross‐referenced with ResearcherID where available, and institutional names (e.g., variations of “University of North Carolina”) were unified to a single canonical form. The time slicing was set to 2000–2024, with a time interval (years per slice) of 1 year. The term “source” was selected from titles, abstracts, and keywords, and the threshold (Top N per slice) was set to 5.

The following is the glossary of bibliometric indicators. Centrality: a metric measuring a node′s role in connecting the network, calculated based on the proportion of shortest paths traversing that node. Burst intensity: a measure reflecting the intensity of a keyword′s steep increase in frequency over a short period, calculated by CiteSpace using the Kleinberg algorithm. Silhouette score: an indicator assessing the quality of cluster analysis; values closer to 1 indicate greater similarity among objects within a cluster.

### 2.3. Sensitivity Analysis of the CiteSpace Threshold

To ensure the robustness of our findings, a sensitivity analysis was conducted on the “Top N” threshold used in CiteSpace. The original analysis employed a threshold of “Top N = 5” per time slice, a common choice in bibliometric studies of this scale that balances the identification of core high‐frequency keywords with the risk of omitting pivotal information due to an overly stringent threshold. We tested the stability of our results against alternative thresholds of “Top N = 3” and “Top N = 8.”

The resulting keyword co‐occurrence networks and cluster structures under these alternative thresholds were highly consistent with the primary analysis. The core research hotspots, such as “primary ciliary dyskinesia,” “mutations,” “cystic fibrosis,” and “genetics,” remained the most prominent nodes across all threshold settings. Furthermore, the thematic clusters (e.g., #1 male infertility and #3 congenital heart disease) demonstrated strong stability. The clustering consistency coefficient between the primary (*N* = 5) and alternative threshold results exceeded 0.9, indicating excellent reliability. These findings confirm that the selection of “Top N = 5” yielded stable and representative results, and the conclusions drawn from our analysis are not sensitive to minor variations in this parameter.

## 3. Analysis and Results

### 3.1. Analysis of Publication Trends

**Figure 1:**
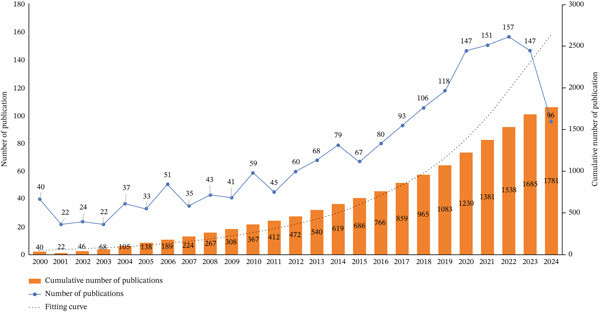
Annual publication trend.

The annual distribution of the 1821 articles retrieved and included from WOS was analyzed to observe the speed, depth, and maturity of the development of this field. The annual publication volume of WOS literature is shown in Figure 1. From 2000 to 2024, the overall publication volume showed a wave‐like increasing trend, with a downward trend emerging in the past 2 years. Between 2000 and 2015, the number of international publications remained stable, showing a slow upward trend, indicating that research in the field of ciliopathies had not yet received widespread attention. From 2015 to 2022, there was a rapid increase in publication volume, rising from 67 articles in 2015 to 157 articles in 2022, with a peak reached in 2022. This shows that in the past decade or so, there has been growing international interest in the field of ciliopathies. Notably, in 2023, there was a temporary decline in WOS publication volume, which may continue to rise in 2024. Overall, research on ciliary diseases has demonstrated an upward trajectory in annual publication output, total citation frequency, and the scale of international collaborative networks, indicating a sustained increase in both academic attention and influence within this field.

### 3.2. Country Co‐Occurrence Analysis

**Figure 2:**
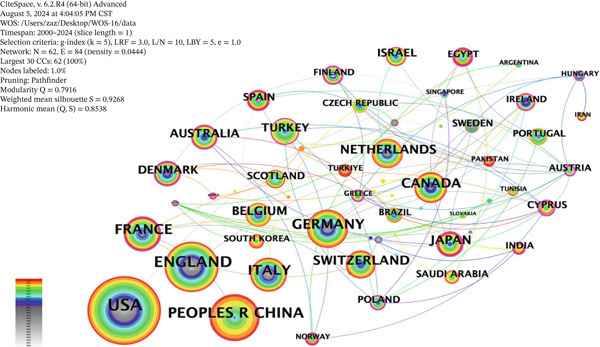
Country co‐occurrence map.

The co‐occurrence network of country publication volumes in ciliopathy research (Figure 2) shows that the larger the radius of a node, the greater the number of publications, and the thicker the outer ring, the higher the centrality. Among the Top 10 countries in the co‐occurrence map of ciliopathy research, France has the highest centrality (0.18), followed by Japan (0.16) and Germany (0.08), indicating that France holds a leading position in ciliopathy research. The United States leads with 533 publications, indicating the largest scale of research output. However, its intermediary centrality is zero, suggesting that within America′s collaborative networks, links between its international partners may be relatively direct, or that the United States does not serve as a pivotal “bridge” connecting research teams from other nations. By contrast, France (centrality 0.18) and Japan (centrality 0.16), despite lower total publication counts, exerted more pronounced hub functions in facilitating transnational collaboration and directing knowledge flows. China ranks third in publication volume in this field, with 218 articles. A total of 62 countries/regions are actively involved in research in this field, with 35 countries/regions having at least 10 publications included in the analysis. Table 1 shows the Top 10 countries/regions in terms of publication volume, with the Top 3 being the United States (*n* = 533), England (*n* = 302), and China (*n* = 218). The cooperation network of these countries/regions is shown in Figure 2. As presented in Table 1, when considering research impact in terms of average citations per publication, the United States, Switzerland, and England lead the field, indicating the strong influence of their research output. The United States, England, and China have broader collaboration networks compared to other countries/regions, indicating that these three countries are at the forefront of ciliopathy research, while other countries have relatively less research activity in this area.

**Table 1:** Top 10 countries ranked by publication volume.

No.	Publication volume	Centrality	Country	Average citations
1	533	0	United States	38.5
2	302	0	England	36.2
3	218	0	People′s Republic of China	29.8
4	182	0.08	Germany	34.1
5	162	0	Italy	32.7
6	160	0.18	France	35.3
7	120	0.06	Canada	33.9
8	114	0	Switzerland	37.6
9	101	0.16	Japan	31.5
10	96	0	The Netherlands	32.9

### 3.3. Institutional Analysis

The Top 10 international research institutions in terms of the publication volume in the field of ciliopathy research, according to WOS data, are listed in the following table. Among them, the University of North Carolina and the University of North Carolina at Chapel Hill are in the first tier, the Institut National de la Sante et de la Recherche Medicale is in the second tier, the Royal Brompton Hospital is in the third tier, and the University of London is in the fourth tier, with other institutions in the fifth tier.

**Figure 3:**
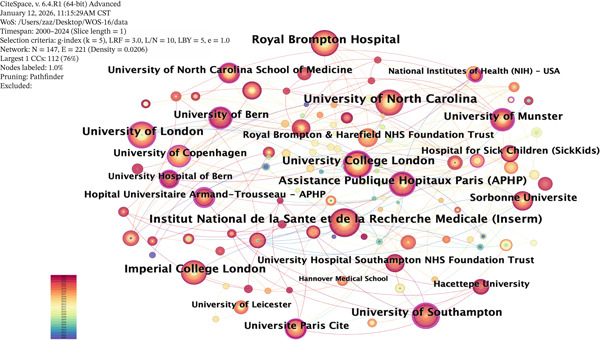
Organization co‐occurrence map.

The included English‐language literature encompasses 144 institutions, with 49 institutions (34%) publishing 10 or more papers and 35 institutions (24.3%) publishing between five and 10 papers. Through the visualization analysis of the cooperation network of institutions publishing in the field of ciliopathies, as shown in Figure 3, 144 nodes and 225 edges were obtained, with a density of 0.0219, indicating a relatively high level of collaboration between institutions. There are 10 institutions with more than 60 publications, including the University of North Carolina (114 articles), University of North Carolina at Chapel Hill (114 articles), Institut National de la Sante et de la Recherche Medicale (100 articles), Royal Brompton Hospital (94 articles), University of London (86 articles), Assistance Publique Hopitaux Paris (83 articles), University College London (82 articles), Imperial College London (78 articles), University of Southampton (74 articles), and University of Munster (61 articles). All the Top 10 institutions have published more than 60 articles, indicating that these institutions are prolific contributors to ciliopathy research. An analysis of the citation impact reveals that the University of North Carolina at Chapel Hill not only leads in publication volume but also in total citation count, underscoring its dominant influence in the field. Other institutions, such as University College London and Imperial College London, also demonstrate high citation rates per publication, reflecting the significant impact of their research. Figure 3 visualizes the macrolevel collaborative landscape among research institutions, highlighting which organizations form the core consortia in ciliopathy research.

### 3.4. Author Analysis

**Figure 4:**
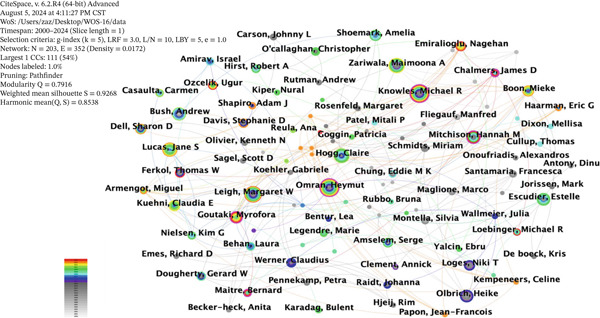
Author collaboration network map.

In the author co‐occurrence network (Figure 4), N = 203, E = 352, and density = 0.0172 indicate that 203 authors were included in the visualization, with 352 collaborative relationships, forming a knowledge network density of 0.0172 for international author collaboration. The size of the nodes and fonts in the network is proportional to the authors′ publication volume. The 1821 English‐language articles include 203 authors, with 15 authors (7.4%) having 20 or more publications and 17 authors (8.4%) having between 10 and 20 publications. The cooperation network visualization (Figure 4) shows that the team led by Omran, Heymut has the highest publication volume and centrality, indicating close collaboration within the team.

The Top 10 authors in terms of publication volume in the field of ciliopathies are listed in Table 2. According to WOS data, Omran, Heymut is in the first tier with 64 papers. Knowles, Michael R. is in the second tier with 58 papers, and Leigh, Margaret W. is in the third tier with 51 papers. Other authors are in the fourth tier. The overall publication and collaboration trends among international authors show that a clear hierarchy has formed, but the overall centrality is low, indicating that collaboration among many authors remained limited. This suggests a need for increased collaboration in ciliopathy research. In contrast, Figure 4 delineates the microlevel collaborative patterns at the individual investigator level, revealing distinct research teams and key leading scholars.

**Table 2:** Top 10 institutions ranked by publication volume.

No.	Publication volume	Centrality	Author
1	64	0.15	Omran, Heymut
2	58	0.12	Knowles, Michael R.
3	51	0.05	Leigh, Margaret W.
4	47	0	Lucas, Jane S.
5	43	0.05	Hogg, Claire
6	39	0.01	Zariwala, Maimoona A.
7	27	0.1	Goutaki, Myrofora
8	26	0.03	Shoemark, Amelia
9	25	0.01	Bush, Andrew
10	24	0.03	Olbrich, Heike

### 3.5. Keyword Co‐Occurrence Analyses

**Figure 5:**
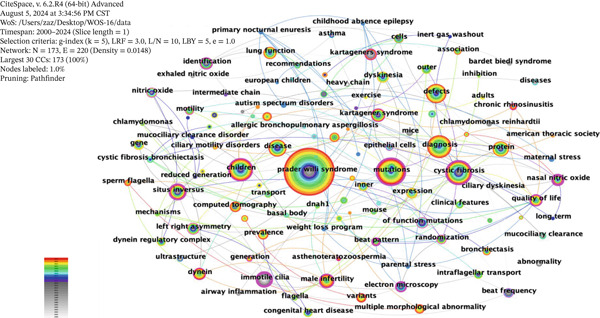
Keyword co‐occurrence map.

Research hotspots are dynamic variables influenced by social development and scientific research. To a certain extent, they reflect the research interests of scholars within specific time periods and indicate the development status of a particular field. Using CiteSpace, a keyword co‐occurrence map for ciliopathy research from 2000 to 2024 was generated. The node type was set to “keyword,” the threshold was set to “Top = 5,” and the time interval was set to 1 year. The keyword co‐occurrence map in the field of ciliopathy research within WOS consists of 173 nodes and 220 edges (Figure 5). The keyword with the highest centrality is “primary ciliary dyskinesia,” indicating a significant focus on PCD in international research. The Top 15 keywords, ranked in descending order of frequency, are “primary ciliary dyskinesia,” “mutations,” “children,” “diagnosis,” “cystic fibrosis,” “defects,” “disease,” “situs inversus,” “protein,” “dyskinesia,” “kartagener syndrome,” “gene,” “expression,” “lung function,” and “left right asymmetry.” These keywords show high co‐occurrence frequency with other keywords. Betweenness centrality is a key indicator for evaluating the importance of network nodes. In the keyword co‐occurrence network for ciliopathy research, keywords with higher centrality include “mutations” (0.46), “cystic fibrosis” (0.46), “situs inversus” (0.41), “children” (0.35), “kartagener syndrome” (0.19), “left right asymmetry” (0.18), and “dyskinesia” (0.1), indicating that mutations, cystic fibrosis, inversions, children, kartagener syndrome, left‐right asymmetry, and dyskinesia are significant research hotspots in the field of ciliopathies. Thus, the keyword co‐occurrence map (Figure 5) effectively outlines the overall intellectual structure and pivotal conceptual hubs of the ciliopathy research landscape.

### 3.6. Keyword Cluster Analysis

**Figure 6:**
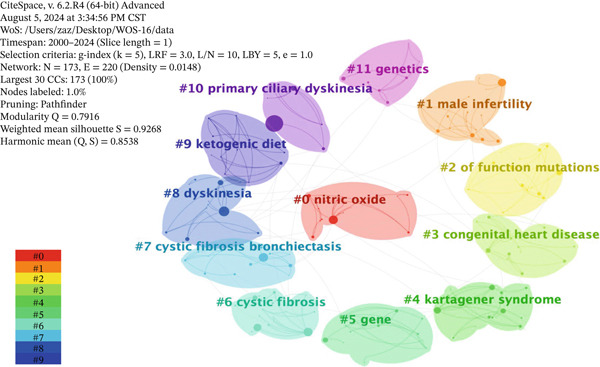
Keyword clustering map.

Building upon the keyword co‐occurrence map, clustering and timeline analysis of keywords can help us more clearly understand the current state of research and identify research hotspots in this field. While Figure 5 reveals the connections between keywords, the clustering analysis in Figure 6 organizes these connections into distinct, automatically generated thematic domains. Cluster analysis is performed using hierarchical clustering based on descending similarity, which intuitively displays the relationships between the articles.

The analysis of high‐frequency keywords reveals 12 main clusters. These clusters are sorted by the number of keywords, and the cluster names are derived from the keywords and the log‐likelihood ratio (LLR) algorithm. The cluster number is inversely proportional to the cluster size. The smaller the cluster number, the larger the cluster size and the more keywords it contains. The silhouette score, which measures the homogeneity within a cluster, is positively correlated with the consistency of the cluster members. A higher silhouette score indicates better homogeneity. The clustering results are shown in Figure 6. The keyword cluster labels include “#0 nitric oxide,” “#1 male infertility,” “#2 of function mutations,” “#3 congenital heart disease,” “#4 kartagener syndrome,” “#5 gene,” “#6 cystic fibrosis,” “#7 cystic fibrosis bronchiectasis,” “#8 dyskinesia,” “#9 ketogenic,” “#10 primary ciliary dyskinesia,” and “#11 genetics.” The silhouette scores for all clusters are greater than 0.8, indicating high consistency and good homogeneity among the clusters. It can be seen that, in the field of ciliopathy research, the primary focus is on functional mutations, congenital heart disease, cystic fibrosis, dyskinesia, PCD, and genetics.

### 3.7. Keyword Timeline Analysis

**Figure 7:**
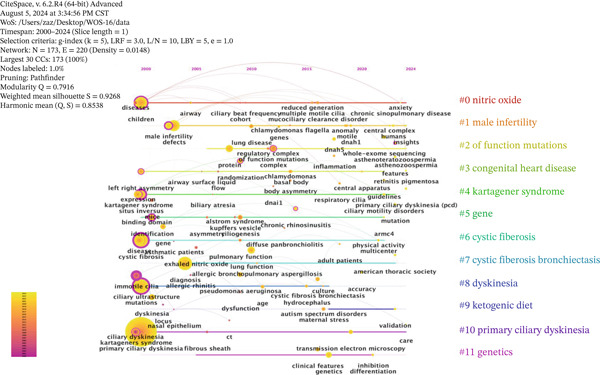
Keyword clustering timeline map.

The evolution of a field refers to its development and changes over time, helping scholars form a correct understanding of its historical progress. To explore the evolution of ciliopathy research, this study utilized a keyword co‐occurrence map with a timeline perspective. The time interval was set to 1 year, and a keyword timeline map of ciliopathy research hotspots from 2000 to 2024 was generated (Figure 7). It helps to more clearly analyze the development and trends in this field.

Vertical analysis of the timeline clustering reveals two main phases: The first stage (2000–2010) was the initial stage of ciliopathy research, which focused on areas such as children, Kartagener syndrome, disease, cystic fibrosis, mutations, and PCD fibrous sheath. The second stage (2010–2024) was the rapid development stage in ciliopathy research, with a shift toward studying mucociliary clearance disorder, functional mutations, maternal stress, transmission electron microscopy, and PCD.

In the horizontal analysis, around 2000, hot research keywords with high frequency were “primary ciliary dyskinesia,” “mutations,” “children,” “diagnosis,” “cystic fibrosis,” and “disease,” with numerous connections between these keywords. This indicates that during this period, research was highly concentrated on PCD, pathogenic mutations, children, disease diagnosis, cystic fibrosis, and related diseases, with the highest research intensity observed in these areas.

### 3.8. Keyword Burst Analysis

**Figure 8:**
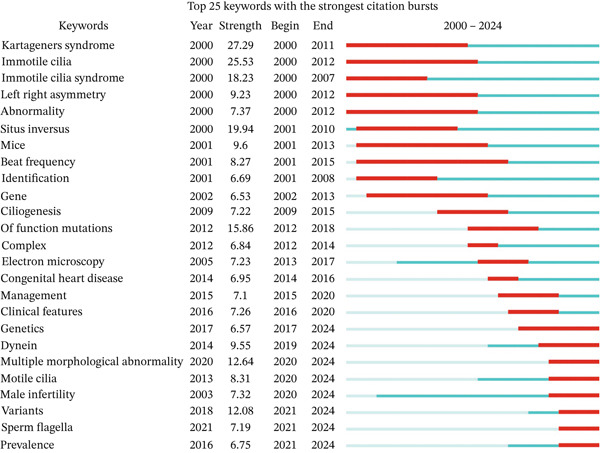
Keyword burst map.

CiteSpace′s keyword burst detection function can analyze keywords that have undergone rapid changes in a short period and can also display the start and end years of the keyword and the burst intensity. This allows us to identify research hotspots from the perspective of keywords both in the past and present. The keyword burst analysis for ciliopathy research is shown in Figure 8. According to burst intensity, the Top 5 burst keywords were “kartageners syndrome,” “immotile cilia,” “situs inversus,” “immotile cilia syndrome,” and “function mutations.” Overall, the research can be divided into three phases. From 2000 to 2013, the research hotspots were mainly focused on Kartagener syndrome, immotile cilia, immotile cilia syndrome, left–right asymmetry, abnormality, situs inversus, and genes, with Kartagener syndrome showing the highest burst intensity (27.29), indicating its significant activity in ciliopathy research. From 2013 to 2020, the focus shifted to ciliogenesis, function mutation, electron microscopy, congenital heart disease, and clinical features, with function mutations showing a high burst intensity (15.86), making it a prominent research area. According to the burst trend analysis up to 2024, current ongoing research hotspots include genetics, dynein, multiple morphological abnormalities, motile cilia, male infertility, variants, sperm flagella, and prevalence. These areas are expected to remain significant research directions in the coming years.

### 3.9. Cross‐Tool Validation With VOSviewer

**Figure 9:**
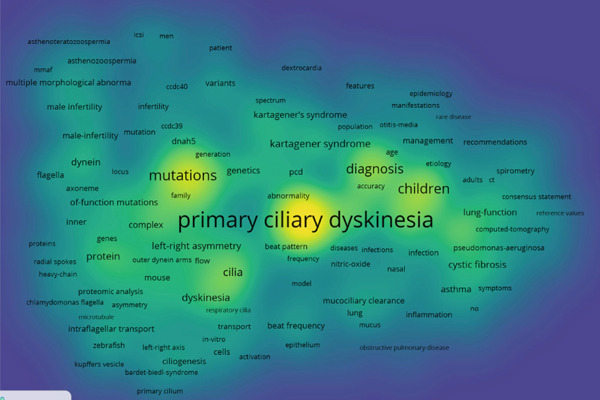
Keyword network density map (VOSviewer).

**Figure 10:**
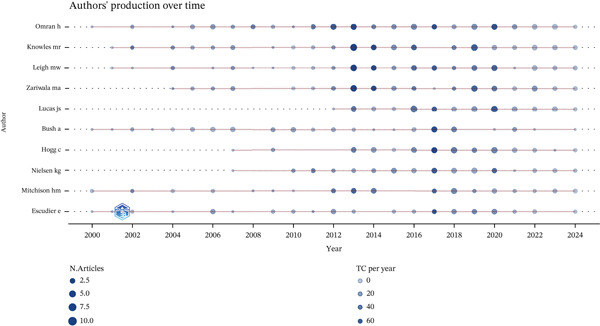
Authors′ production over time map (Bibliometrix).

**Figure 11:**
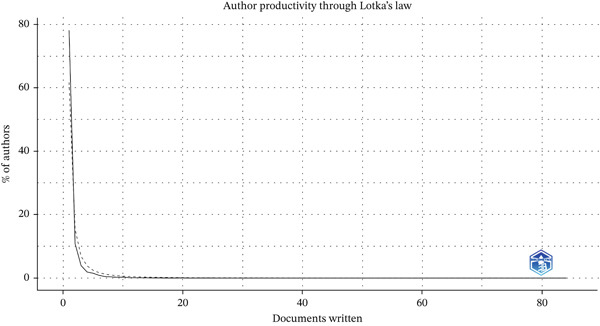
Author productivity analysis map (Bibliometrix).

To address potential tool‐specific biases and enrich the analytical perspective, we replicated the keyword co‐occurrence analysis using VOSviewer software (Version 1.6.20). The same dataset of 1821 articles was imported into VOSviewer, employing a binary counting method for the keyword network. The resulting network map (Figure 9) exhibited a high degree of structural consistency with the network generated by CiteSpace (Figure 5). Core research domains, such as “PCD,” “mutations,” and “cystic fibrosis,” consistently emerged as the largest and most centrally connected nodes in both visualizations. Using the Bibliometrix tool to analyze authors′ publication trends over the years (Figure 10), the results indicate that Omran, Knowles, Leigh, and Zariwala produced a higher volume of papers in 2013, while Bush, Hogg, and Nielsen achieved their peak output in 2017. Analysis of author productivity using Lotka′s law (Figure 11) reveals that authors publishing a large number of papers constitute only a very small minority of all authors. The vast majority publish only one or a very small number of papers throughout their careers. This cross‐tool validation reinforces the robustness of our primary findings regarding the intellectual structure and key research hotspots in the ciliopathy field.

## 4. Discussions

Ciliopathies are a type of genetic disease caused by structural and/or functional abnormalities of cilia. It primarily affects multiple systems, including the kidneys, lungs, and nasal epithelium [16, 17]. Cilia play important physiological functions in the kidney, including participating in urine concentration, electrolyte balance, and acid–base regulation. Ciliary dysfunction often leads to kidney diseases [18]. With the development of high‐throughput sequencing technology, researchers have gradually revealed the genetic basis of ciliopathies. For example, mutations in certain genes, such as the disruption of Dhcr7 and Insig1/2, can lead to abnormal kidney development and function, manifested as kidney disease [10]. The main function of cilia in the lungs is to remove foreign matter and pathogens from the respiratory tract and maintain the cleanliness and patency of the respiratory tract. Abnormal ciliary function can lead to the occurrence of lung diseases, such as PCD [19]. For diseases with abnormal ciliary function, such as PCD, researchers are exploring new treatments such as gene therapy. By repairing or replacing defective genes, the normal function of cilia can be restored, thereby improving the patient′s clinical symptoms [20]. Cilia in the nasal epithelium are essential for maintaining the cleanliness and patency of the nasal cavity. Abnormal ciliary function can lead to the occurrence of nasal diseases, such as sinusitis [21]. Studies have shown that ciliary dysfunction is one of the important causes of sinusitis. Impaired ciliary function leads to defective clearance of nasal secretions, thereby causing nasal diseases such as sinusitis [22]. This study, to our knowledge, represents one of the first dedicated bibliometric analyses to map the knowledge structure and research trends in the ciliopathies field systematically. By employing CiteSpace, we have moved beyond a qualitative summary to provide a quantitative and visual synthesis of two decades of research. Our analysis not only corroborates known research areas but also objectively identifies the evolution of research foci (e.g., the shift from syndromic descriptions like Kartagener syndrome to molecular mechanisms like functional mutations and ciliogenesis), reveals underappreciated collaborative patterns (e.g., the high centrality of France despite lower volume and the isolated clusters within the author network), and highlights emergent frontiers (e.g., genetics, dynein, and male infertility) that are poised to define future research. This systematic mapping provides a significant advance by offering a foundational reference and an analytical framework that can help newcomers navigate the field, assist funding bodies in identifying promising areas, and stimulate collaborative opportunities among researchers from different regions and disciplines.

From 2000 to 2024, the overall publication volume exhibits a wave‐like upward trend, with a recent decline in the past 2 years. Between 2000 and 2015, the number of international publications remained relatively stable, showing a slow upward trend. However, from 2015 to 2022, the publication volume increased rapidly, rising from 67 articles in 2015 to 157 articles in 2022, reaching its peak in 2022. This rapid growth in publications has propelled the swift development of ciliopathy research, shifting the focus during this period toward areas such as mucociliary clearance disorder, functional mutations, maternal stress, transmission electron microscopy, and PCD. Functional mutations and PCD have emerged as key research hotspots. This shift indicates that ciliopathy research has transitioned from being relatively overlooked to becoming widely recognized. An analysis of international publication trends indicates that ciliopathy research is gradually increasing overall, signifying its rising importance in the scientific community.

In the field of ciliopathy research, the United States, England, and China are leading, not only in publication volume but also in the breadth of their collaborations. Researchers from the United States and China have cooperated to prove that the increase in DNA damage response is not inherent to ciliary defects and that Relitin plays a role in multiple processes in vertebrate development, including ciliary specificity and ciliary independence [23]. Researchers from the United States and England have jointly pointed out that the diagnosis of PCD is complex and requires the integration of medical history, clinical manifestations, imaging, functional and structural analysis of motile cilia, and even genetic testing to make a clear diagnosis [24]. Countries such as France, Germany, and Japan have also shown a high level of research activity in this field, indicating that these countries place significant emphasis on ciliopathy research. Furthermore, international cooperation and exchange play a crucial role in advancing the depth of ciliopathy research. In the institutional cooperation network, the University of North Carolina and its subsidiary institution, the University of North Carolina at Chapel Hill, have performed outstandingly, ranking among the top in publication volume. In addition, institutions such as the Institut National de la Sante et de la Recherche Medicale and the Royal Brompton Hospital have also played an important role in this field. The research of these institutions has not only promoted the development of the basic theory of ciliopathies but also provided critical evidence for the diagnosis and treatment of diseases.

In the author cooperation network, scholars such as Omran, Heymut and Knowles, Michael R. have become central figures in this field. These researchers and their teams have not only published numerous high‐quality papers but also advanced ciliopathy research through close collaboration and communication. For example, Omran, Heymut and Knowles, Michael R. have cooperated in studying the role of cilia in the formation of hydrocephalus [25], novel pathogenic variations in DNAH5 associated with the clinical and genetic spectrum of PCD in the Arab population [26], and the current and future treatment of PCD. Among them, in the current and future treatment of PCD, the researchers detailed the clinical research on PCD conducted so far and explored precision medicine methods for PCD, including gene therapy, mRNA transcription, and read‐through therapy [27]. However, overall, the author cooperation network in the field of ciliopathies remains somewhat loose, with many authors collaborating infrequently. Therefore, more scholars should be encouraged to join the research on ciliopathies, strengthen cross‐disciplinary and cross‐field cooperation and communication, and jointly promote the prosperity and development of this field.

From the analysis of keyword co‐occurrence, clustering, and timeline, prior to 2010, the research on ciliopathies mainly focused on Kartagener syndrome, cystic fibrosis, mutation, and PCD. In recent years, the research hotspots have shifted toward functional mutations, congenital heart disease, cystic fibrosis, dyskinesia, PCD, and genetics. Our keyword salience and timeline analysis reveal that the term “Kartagener syndrome” exhibited its most pronounced outbreak intensity between 2000 and 2013, followed by a subsequent decline in its relative frequency of use. This bibliometric trend may be driven by two concurrent factors. First, reflecting the intrinsic evolution of scientific research, advancements in high‐throughput sequencing and functional genomics have shifted the research paradigm from broad descriptions of clinical syndromes toward detailed investigations of specific disease‐causing genes, molecular mechanisms, and cellular phenotypes. Consequently, more descriptive and etiologically directed terminology, such as “PCD” or nomenclature based on specific genetic defects, has gained increasing prevalence in scientific discourse. Secondly, regarding external regulatory shifts in medical nomenclature, as the reviewers astutely noted, there exists a growing international trend within the medical and genetics communities to move away from eponyms. Numerous academic bodies and nomenclature guidelines favor descriptive terms that directly reflect a disease′s pathophysiology, anatomical location, or genetic characteristics, thereby enhancing precision, universality, and cultural neutrality. Consequently, the widespread adoption of “PCD” as a descriptive term aligns with this broader disciplinary shift in norms. In summary, the interplay between intrinsic scientific refinement and extrinsic naming standardization has collectively reshaped the relative significance of the historical term “Kartagener syndrome” in the literature. This shift has propelled “PCD” and associated molecular terminology to become the mainstream vocabulary in contemporary research discourse. This is exemplified by the fact that, as our cluster analysis shows (#10 primary ciliary dyskinesia), there is no single gold standard, and diagnosis relies on a combination of tests [28]. Furthermore, our burst detection (Figure 8) identifies “genetics” and “variants” as strong, ongoing research frontiers. This data‐driven insight aligns with the growing emphasis on genetic testing and the discovery of new pathogenic genes (now over 50 [28]), directly foreshadowing the application of novel genomic technologies. For instance, the functional genomic CRISPR‐Cas9 screen mentioned [29] serves as a prime example of the kind of research that is propelling this trend identified by our analysis. This screen not only provides an unbiased tool for ciliopathies classification but also validates the link between ciliary signaling and congenital heart disease—another prominent hotspot (#3 congenital heart disease) identified in our clustering results. These research hotspots not only reflect the main directions of current ciliopathy research but also provide important references for the diagnosis and treatment of ciliopathies. For example, a study used phenotypic assessments derived from electronic health records (EHR) to diagnose ciliopathies using three online‐accessible rare disease diagnostic support systems (DSS) [30]. In recent years, there has been an increasing number of studies on treatment strategies for ciliopathies, mainly including the use of antimicrobial drugs to control infection and oxygen therapy to relieve symptoms such as dyspnea. Studies have shown that targeted gene replacement can restore the morphology and function of olfactory cilia in olfactory sensory neurons and further reconstruct odor‐guided behavior in animals [31]. Some studies have found that pharmacological intervention of the FGF‐PTH axis can be used as a potential treatment for craniofacial ciliopathies [32]. In addition, keyword burst analysis also revealed keywords that have undergone significant changes in ciliopathy research in a short period of time. Keywords such as “kartageners syndrome” and “immotile cilia” became research hotspots in a specific period, which may be closely related to the research progress and clinical needs at that time. The ongoing research on keywords such as “genetics,” “dynein,” and “multiple morphological abnormality” indicates that these areas will continue to be the focus of ciliopathy research in the next few years.

Synthesizing the key findings from our bibliometric analysis, we can project several data‐driven future directions for ciliopathy research. The strong and ongoing burst strength of keywords such as “genetics,” “dynein,” “variants,” and “male infertility” unequivocally signals that the exploration of the genetic architecture and molecular mechanisms of ciliopathies will remain a central theme. This genetic focus naturally extends to the application of advanced gene‐editing technologies like CRISPR‐Cas9 for both functional validation and potential therapeutic exploration. Concurrently, the persistent significance of clusters related to “congenital heart disease” (#3) and the emergence of “male infertility” (#1) as a major cluster highlight that the pathogenesis and development of targeted interventions for these high‐impact clinical phenotypes will continue to be a priority. Furthermore, the timeline view and burst detection reveal a growing emphasis on specific ciliary subtypes, particularly “motile cilia” and “sperm flagella,” guiding the field toward more precise mechanistic studies. Future approaches combining whole‐exome sequencing with ultrastructural analysis of motile cilia or sperm flagella will enable precise etiological diagnosis and classification for each patient. Furthermore, by identifying specific signaling pathways and key proteins in motile cilia or sperm flagella, small‐molecule drugs can be designed to enhance ciliary beating function or regulate sperm motility. Therefore, based on our objective data, future research is poised to deepen its focus on (1) advanced genetics and gene editing, driven by the persistent burst strength of genetic keywords; (2) mechanistic studies of ciliary formation and function, encompassing structural components like dynein and specific motile cilia systems; and (3) phenotype‐targeted therapies, addressing both long‐standing and emerging clinical challenges identified in our cluster analysis, such as congenital heart disease and infertility. Finally, the relatively low overall centrality in the author collaboration network (Figure 4) underscores the need for enhanced interdisciplinary and international cooperation to translate these insights into clinical advancements.

## 5. Limitations and Future Directions

It is important to acknowledge the limitations of this study. Firstly, our analysis was restricted to English‐language literature retrieved from the WOS database. This approach may have resulted in the omission of valuable research published in other languages (e.g., Chinese, German, or Japanese) or regional databases, potentially introducing a selection bias and limiting the comprehensiveness of our findings. Secondly, this study primarily utilized CiteSpace for its analytical framework. Although we conducted a cross‐validation of the keyword co‐occurrence network using VOSviewer, which confirmed the robustness of the main research themes, a more comprehensive integration of diverse bibliometric tools (e.g., Bibliometrix for performance analysis) in future research could uncover additional insights and offer complementary perspectives.

Beyond these methodological considerations, our findings also point to substantive gaps in the ciliopathy research landscape that present opportunities for future inquiry. Firstly, the strong burst strength of genetic keywords underscores that, despite significant progress, the full genetic architecture of ciliopathies remains incompletely characterized, and the functional validation of identified variants using tools like CRISPR‐Cas9 represents a major future frontier. Secondly, the identification of high‐impact clinical phenotypes like congenital heart disease and male infertility as persistent clusters highlights a translational gap; while these associations are well‐documented, the development of targeted, mechanism‐based therapies for these specific manifestations remains an unmet need and represents a critical direction for future research.

This study systematically revealed the hotspots and development trends in ciliopathy research through bibliometric and CiteSpace visualization analyses. As a type of genetic disease caused by structural and/or functional abnormalities of cilia, ciliopathies have increasingly become a hot topic in biomedical research in recent years. The research hotspots are mainly concentrated on functional mutations, congenital heart disease, cystic fibrosis, dyskinesia, PCD, and genetics. Analysis indicates that research in the field of ciliary diseases internationally exhibits a wave‐like upward trend, with institutions in the United States, the United Kingdom, and China leading in output. The outstanding performance of these institutions is often intrinsically linked to their core research teams. Future collaborative efforts should focus concurrently on establishing platforms between institutions and deepening networks among core researchers. Through co‐occurrence analysis of keywords, cluster analysis, timeline analysis, and outbreak analysis, this study not only captures the historical development of ciliopathy research but also projects possible future directions, such as genetics, dynein, and motile cilia, providing valuable references for further investigations in the field.

## Author Contributions

Qian Dong and Jinhao Zhu: conceptualization, methodology, software, investigation, visualization, writing—original draft, and investigation. Huan Xu and Jiang Liu: conceptualization, writing—original draft, funding acquisition, and supervision. Qian Dong and Jinhao Zhu contributed equally to this work.

## Funding

This study was funded by the Doctor Development Foundation of Lihuili Hospital (2022BSKY‐XH) and the Key Cultivating Discipline of Lihuili Hospital (2022‐P06).

## Disclosure

All the authors in this study agreed to the publication of the manuscript.

## Ethics Statement

The authors have nothing to report.

## Conflicts of Interest

The authors declare no conflicts of interest.

## Data Availability

All data generated or analyzed during this study are included in this article. Further inquiries should be directed to the corresponding author.

## Appendix A Detailed Search Strategy for Web of Science

The following search query was executed on the Web of Science Core Collection on July 27, 2024, to retrieve literature for this bibliometric analysis. The strategy combines Medical Subject Headings (MeSH) terms, synonyms, abbreviations, and emerging keywords related to ciliopathies using the Boolean “OR” operator within the topic field (TS).

Search query:

TS=(

(“ciliopath”) OR

(“primary ciliary dyskinesia” OR “PCD”) OR

(“kartagener syndrome”) OR

(“ciliary motility disorder” OR “immotile cilia syndrome”) OR

(“ciliary disorder” OR “ciliary dysfunction”) OR

(“motile cilia defect”) OR

(“sperm flagella variant∗”)

)

Refinements:

Indexes: SCI‐EXPANDED, SSCI, A&HCI, CPCI‐S, CPCI‐SSH, and ESCI.

Time span: all years (2000–2024, as of the search date).

Document types: article or review.

Language: English.
